# Triptolide potentiates lung cancer cells to cisplatin-induced apoptosis by selectively inhibiting the NER activity

**DOI:** 10.1186/s40364-015-0043-2

**Published:** 2015-07-09

**Authors:** Gan Wang, Xing Wang, Xiaoxin Xu

**Affiliations:** Institute of Environmental Health Sciences, Wayne State University, 259 Mack Avenue, Detroit, MI 48201 USA; Array Bridge Inc., St. Louis, MO USA

**Keywords:** Cisplatin, Triptolide, Lung cancer cells, Apoptosis, NER, Inhibition of NER, Potentiation of lung cancer cells

## Abstract

**Background:**

Cisplatin and many other platinum-based compounds are important anticancer drugs that are used in treating many cancer types. The development of cisplatin-resistant cancer cells, however, quickly diminishes the effectiveness of these drugs and causes treatment failure. New strategies that reverse cancer cell drug resistance phenotype or sensitize cancer cells to these drugs, therefore, need to be explored in order to improve platinum drug-based cancer treatment. Triptolide is a bioactive ingredient isolated from *Tripterygium wilfordii*, a Chinese herbal medicine. Triptolide binds to the TFIIH basal transcription factor and is required for both transcription and nucleotide excision repair (NER), a DNA repair pathway involved in repairing DNA damage generated by the platinum-based anticancer drugs.

**Methods:**

Caspase-3 activation and cell growth inhibition assays were used to determine the effect of triptolide on cisplatin-induced apoptosis and cell growth in lung cancer cells. Real time PCR, immunoblotting, and expression of reef coral red protein were used to determine a mechanism through which the presence of triptolide increased cisplatin-induced apoptosis of the lung cancer cells.

**Results:**

Our caspase-3 activation studies demonstrated that the presence of low-levels of triptolide greatly increased the cisplatin-induced apoptosis of HTB182, A549, CRL5810, and CRL5922 lung cancer cells. The results of our cell growth inhibition studies revealed that the presence of low-levels triptolide itself had little effect on cell growth but greatly enhanced cisplatin-induced cell growth inhibition in both A549 and HTB182 cells. The results of our reef coral-red protein reporter expression studies indicated that the presence of low-levels triptolide did not affect expression of the reef coral-red protein from pDsRed2-C1 plasmid but greatly inhibited expression of the reef coral-red protein from cisplatin-damaged pDsRed2-C1 plasmid DNA in A549 cells. In addition, the results of our protein phosphorylation studies indicated that the presence of low-levels triptolide caused a decrease for cisplatin-induced CHK1 phosphorylation at Ser^317/345^ but an increase for cisplatin-induced ATM phosphorylation at Ser^1981^ in both HTB182 and A549 cells.

**Conclusion:**

The results of our studies suggest that the presence of low-levels of triptolide potentiates lung cancer cells to cisplatin treatment by selectively inhibiting NER activity, resulting in an increase in apoptosis of the lung cancer cells.

## Introduction

Cisplatin and many other platinum-derived chemical compounds are important anticancer drugs that have been used in treating many cancer types, including lung cancer [1]. The effectiveness of these drugs, however, often diminishes as cancer cells quickly develop their resistance to the drugs, leading to treatment failure [2]. Novel strategies that can potentiate cancer cells to these drugs need to be explored in order to improve the effectiveness of these drugs in cancer treatment.

Platinum-based anticancer drugs, including cisplatin, execute their anticancer activities mainly by generating DNA damage (*e.g.,* intra- and inter-strand crosslinks) and promoting DNA damage-induced cell cycle arrest and apoptosis [3–6]. However, cancer cells can reduce or eliminate the cytotoxicity of platinum-based anticancer drugs by repairing the platinum DNA damage using nucleotide excision repair (NER), a DNA repair pathway that is required for removing DNA damage generated by many environmental carcinogens and therapeutic drugs [3]. In addition, cancer cells can also reduce the cytotoxicity of the platinum-based drugs by altering the membrane permeability to reduce cellular uptake in these drugs [1, 2]. The effectiveness of platinum-based anticancer treatment would be greatly improved if the NER activity can be inhibited in cancer cells.

Triptolide is a bioactive ingredient isolated from *Tripterygium wilfordii*, a traditional Chinese herbal medicine [7]. Clinical studies reveal that triptolide possesses both anti-inflammation and immuno-suppressor activities [7–9]. Biochemical studies demonstrated that triptolide binds specifically to XPB, a component of the TFIIH basal transcription factor, and inhibits its ATPase activity [10]. Recent studies reveal some anti-tumor activities of triptolide [11–13] by promoting a CDK7-dependent RNA polymerase II (Pol II) degradation and global transcription inhibition [14]. Triptolide is known to interfere with a number of transcription factors, including p53 [15], NF-κB [16], nuclear factor of activated T-cells (NFAT) [16], and heat shock factor protein 1 (HSF-1) [17]. The clinical application of triptolide is limited by its severely adverse side effects, especially at high concentrations, likely through its global transcription inhibition effect. Given the important roles of TFIIH in both transcription and NER processes, however, it is possible that triptolide may be used to potentiate cancer cells to the platinum-based cancer treatment by disrupting the NER pathway and increasing apoptosis.

In our recent studies, we investigated the effect of low-levels of triptolide on cisplatin-induced apoptosis of lung cancer cells. Using A549, HTB182, CRL5810, and CRL5922 lung cancer cells, results obtained from our caspase-3 activation studies demonstrated that the presence of low-levels of triptolide caused a great increase in cisplatin-induced caspase-3 activation in these lung cancer cells whereas the presence of triptolide or cisplatin alone had little effect in activating the caspase-3 in these lung cancer cells. The results of our cell proliferation studies revealed that the presence of low-levels triptolide only had moderate or no effect on cell proliferation but significantly increased cisplatin-induced cell growth inhibition in both A549 and HTB182 cells*.* The results of our reef coral-red protein expression studies revealed that the presence of triptolide had little effect on the expression of reef coral-red protein from pDsRed2-C1 plasmid but great effect on inhibiting the expression of the reef coral-red protein from cisplatin-damaged pDsRed2-C1 plasmid in A549 lung cancer cells. In addition, the results of our protein phosphorylation studies indicated that the presence of triptolide reduced cisplatin-induced CHK1 phosphorylation at Ser^317/345^ but increased cisplatin-induced ATM phosphorylation at Ser^1981^ in both A549 and HTB182 lung cancer cells. These results suggest that the presence of low-levels of triptolide potentiates lung cancer cells to cisplatin-induced apoptosis by inhibiting the NER activity, resulting in a great increase in apoptosis of these lung cancer cells.

## Results

### The presence of low-levels triptolide had little effect on cell proliferation or gene transcription in A549 and HTB182 lung tumor cells

**Fig. 1:**
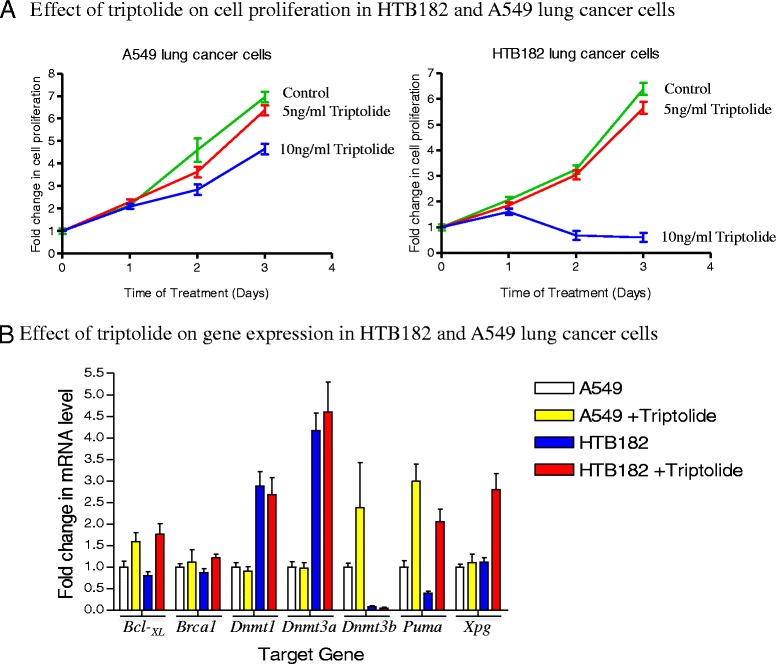
The effect of triptolide on cell proliferation and gene transcription in both A549 and HTB182 lung tumor cells. For cell growth study, cells were collected at 24, 48, and 72 h after the triptolide treatment. For gene transcription study, cells treated with triptolide (5 ng/ml for HTB182 and 10 ng/ml for A549 cells) for 20 h and total RNA isolated from the cells were analyzed by real time PCR assay

To determine if the presence of low-levels of triptolide has any effect on cell growth, we first performed a cell proliferation study. Both A549 and HTB182 lung tumor cells were seeded onto 100 mm dishes with the same number of cells. The cells were either left untreated or treated with 5 ng/ml and 10 ng/ml triptolide (14nM and 28nM) respectively. At different time points (0, 24, 48, and 72 h), cell numbers were counted for both the untreated and triptolide-treated dishes (Fig. 1a). The results of our cell proliferation studies revealed that the presence of 5 ng/ml triptolide had little effect on inhibiting cell proliferation in both HTB182 and A549 cells; however, the presence of 10 ng/ml has a limited effect in inhibiting cell proliferation in A549 cells but great effect in inhibiting cell proliferation of HTB182 cells (Fig. 1a). Although the mechanism for this inhibition effect was unknown, it was likely that the global transcription inhibition effect of triptolide contributed to this increased cell proliferation inhibition in HTB182 cells.

To further determine the effect of low-levels of triptolide on gene transcription, we performed a reverse transcription-based RNA quantification (real time PCR) study to determine the mRNA level of several genes involved in DNA repair, DNA methylation, and apoptosis, in both untreated and triptolide-treated A549 and HTB182 lung tumor cells (Fig. 1b). The results of our real time PCR studies indicated that the presence of triptolide caused increased transcription in most of the tested genes. Therefore, no global transcription inhibition effect was detected in these lung tumor cells treated with low-levels of triptolide. All of our studies, therefore, were done using 10 ng/ml triptolide for A549 and 5 ng/ml triptolide for HTB182 lung tumor cells.

### The presence of low-levels of triptolide resulted in a great increase of cisplatin-induced caspase-3 activation in lung tumor cells

**Fig. 2:**
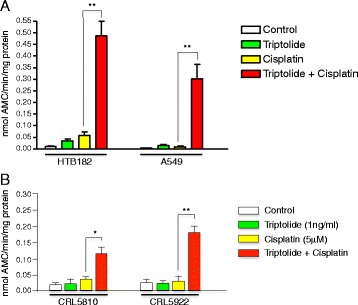
The effect of triptolide on cisplatin-induced caspase-3 activation in various lung cancer cells. **a** The effect of triptolide on cisplatin-induced caspase-3 activation in A549 and HTB182 lung tumor cells. The cells were treated with triptolide (10 ng/ml for A549 and 5 ng/ml for HTB182), cisplatin (5 μM), or a combination of both triptolide and cisplatin (5 μM) for 36 h and caspase-3 activity was determined. **b** The effect of triptolide on cisplatin-induced caspase-3 activation in CRL5810 and CRL5922 lung tumor cells. The cells were treated with triptolide (1 ng/ml), cisplatin (5 μM), or a combination of both triptolide (1 ng/ml) and cisplatin (5 μM) for 26 h and caspase-3 activity was determined. The caspase-3 activity was determined using the Ac-DEVD-AMC as a substrate and measured as nmol AMC/min/mg protein. The *p* value <0.01 was considered statistically significant in this study (*p value < 0.01; **p value < 0.001)

Although triptolide has been demonstrated in its anticancer activities [11–13], most of these anticancer activities were observed at relatively high levels of triptolide presumably through binding to TFIIH and causing global transcription inhibition. Unfortunately, high levels of triptolide lead to severely adverse effects, which limit its potential implication on cancer treatment. Interestingly enough, the TFIIH is also involved in the NER process [3, 18]. The NER process repairs the cisplatin DNA damage and eliminates the cytotoxic effect of cisplatin. Therefore, it is possible that triptolide may be used as a chemo-sensitizer to potentiate cancer cells to platinum-based cancer treatment by disrupting the NER activity and increasing the apoptosis event. To explore this possibility, we first determined the effect of low-levels of triptolide on cisplatin-induced caspase-3 activation of A549 and HTB182 lung tumor cells. Both A549 and HTB182 cells were treated with cisplatin (5 μM) or triptolide (10 ng/ml for A549 and 5 ng/ml for HTB182) alone or a combination of both cisplatin and triptolide for 36 h and the caspase-3 activity was determined (Fig. 2a). As controls, caspase-3 activity was also determined from the untreated A549 and HTB182 cells (Fig. 2a). The results of our caspase-3 activation studies revealed that treatment of the A549 and HTB182 cells with cisplatin or triptolide alone had little effect on activation of the caspase-3 in these cells (Fig. 2a). When treated with both cisplatin and triptolide, however, a great increase in caspase-3 activity was observed in both A549 and HTB182 cells (Fig. 2a).

To determine whether the effect of low-dose triptolide in sensitizing lung cancer cells to cisplatin treatment is limited to HTB182 and A549 lung tumor cells and if this effect also exists in other lung tumor cells, we further studied the effect of low-dose triptolide on cisplatin-induced caspase-3 activation in CRL5810 and CRL5922 lung tumor cells and a lower-dose triptolide (1 ng/ml) was used in the study (Fig. 2b). Interestingly, the presence of lower dose triptolide (1 ng/ml) also was able to potentiate these lung tumor cells to cisplatin-induced caspase-3 activation in both CRL5810 and CRL5922 lung tumor cells (Fig. 2b).

**Fig. 3:**
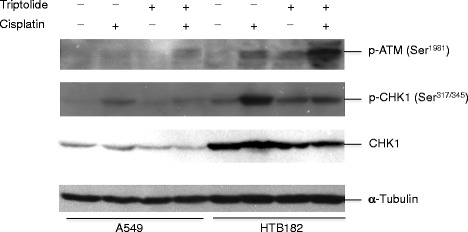
The effect of triptolide on cisplatin-induced CHK1 phosphorylation at Ser^317/345^ and ATM phosphorylation at Ser^1981^ in A549 and HTB182 lung tumor cells. The A549 and HTB182 lung tumor cells were treated with triptolide (10 ng/ml for A549 and 5 ng/ml for HTB182), cisplatin (5 μM), or a combination of triptolide and cisplatin for 20 h. The cells were collected and 30 μg of total protein from each cell lysate was used in the western blotting

Together, these results suggested that the presence of low-level triptolide indeed increased the cisplatin-induced apoptosis in these lung tumor cells.

### The presence of low-levels of triptolide caused an increase of cisplatin-induced ATM phosphorylation at Ser^1981^ and a decrease of cisplatin-induced CHK1 phosphorylation at Ser^317/345^ in both A549 and HTB182 lung cancer cells

To further define a mechanism through which the presence of triptolide causes an increase of cisplatin-induced caspase-3 activation in A549 and HTB182 lung cancer cells, we determined the effect of triptolide on cisplatin-induced CHK1 phosphorylation at Ser^317/345^ and ATM phosphorylation at Ser^1981^ (Fig. 3). The CHK1 phosphorylation at Ser^317/345^ results in an increased cell survival rate from DNA-damaging treatment [19–22] whereas the ATM phosphorylation at Ser^1981^ indicates the presence of DNA double-stranded breaks (DSBs) [23]. The results of our protein phosphorylation studies revealed that the presence of triptolide caused a decrease of cisplatin-induced CHK1 phosphorylation at Ser^317/345^ and an increase of cisplatin-induced ATM phosphorylation at Ser^1981^ in both A549 and HTB182 cells (Fig. 3). Therefore, it is likely that inhibiting the CHK1 phosphorylation and increasing the ATM phosphorylation play important roles in the mechanism through which the presence of low-levels of triptolide potentiates both A549 and HTB182 lung cancer cells to cisplatin-induced apoptosis.

### The presence of low-levels of triptolide inhibited expression of reef coral red protein from cisplatin-damaged pDsRed2-C1 plasmid in A549 lung tumor cells

**Fig. 4:**
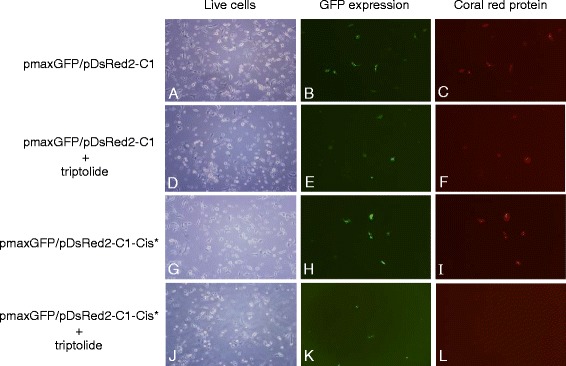
The effect of triptolide on reef coral red and GFP proteins expressions in A549 lung tumor cells. The undamaged or cisplatin-damaged pDsRed2-C1 plasmid (pDsRed2-C1-Cis*) was co-transfected with pmaxGFP plasmid DNA into A549 cells. The transfected cells were cultured in the presence or absence of triptolide (10 ng/ml) for 24 h and expressions of both reef coral red and GFP proteins were detected by fluorescence microscope using excitation/emission lights with wavelengths of 563 nm/582 nm and 475 nm/505 nm respectively for reef coral red and GFP proteins. The light image of the same view was also documented for visualization of the live cells

To further define the mechanism through which the presence of low-level triptolide caused an increase of cisplatin-induced apoptosis in these lung tumor cells, we investigated whether the presence of low-level triptolide inhibited both transcription and the NER process or only one of these events using A549 lung tumor cells. The cisplatin-damaged pDsRed2-C1 plasmid DNA was transfected into A549 cells for DNA repair and expression of reef coral red protein in the presence or absence of triptolide. As a positive control, the undamaged pDsRed2-C1 plasmid was also transfected into A549 cells in a parallel experiment for expression of the reef coral red protein in the presence or absence of triptolide. As an internal control, pmaxGFP plasmid DNA, which carried a CMV promoter-driven green fluorescent protein (GFP) gene, was co-transfected with pDsRed2-C1 plasmid DNA into A549 lung cancer cells for expression of the GFP protein in the presence or absence of triptolide. Expressions of both reef coral red and GFP proteins were visualized using fluorescence microscope (Fig. 4). When undamaged pDsRed2-C1 plasmid was transfected into A549 lung cancer cells, the reef coral red protein was highly expressed in the transfected cells with or without triptolide (Fig. 4f vs 4c). The reef coral red protein was also highly expressed in the cisplatin-damaged pDsRed2-C1 plasmid-transfected A549 cells in the absence of triptolide (Fig. 4i). In the presence of triptolide, however, expression of the reef coral red protein from the cisplatin-damaged pDsRed2-C1 plasmid was greatly diminished in A549 cells (Fig. 4l vs 4i). As an internal control, expression of the GFP protein from pmaxGFP plasmid was not affected by the presence of triptolide, even when expression of the reef coral red protein from cisplatin-damaged pDsRed2-C1 plasmid DNA was greatly inhibited in A549 cells by triptolide in the same dish as demonstrated in our study (Fig. 4k vs 4l). These results suggest that inhibiting NER process plays an important role in the mechanism through which the presence of low-level triptolide reduces reef coral red protein expression from the cisplatin-damaged pDsRed2-C1 plasmid in A549 lung cancer cells.

## Discussion

In these studies, we investigated the effect of low-level triptolide on cisplatin-induced apoptosis and the involved mechanism. The results of our caspase-3 activation studies demonstrated that the presence of low-levels of triptolide greatly increased cisplatin-induced caspase-3 activation in A549, HTB182, CRL5810, and CRL5922 lung tumor cells. The results of our cell proliferation studies revealed that the presence of low-levels of triptolide had little effect on cell proliferation of A549 and HTB182 lung tumor cells. The results of our protein phosphorylation studies indicated that the presence of triptolide caused a decrease in cisplatin-induced CHK1 phosphorylation at Ser^317/345^ and an increase in cisplatin-induced ATM phosphorylation at Ser^1981^ in both A549 and HTB182 lung tumor cells. The results of our reef coral red protein expression studies revealed that the presence of low-level triptolide only inhibited expression of the reef coral red protein from the cisplatin-damaged but not the undamaged pDsRed2-C1 plasmid in A549 lung tumor cells. All of these results suggest that the presence of low-levels triptolide potentiates lung cancer cells to cisplatin treatment by inhibiting NER activity and enhancing cisplatin DNA damage-induced apoptosis.

The results of our caspase-3 activation studies demonstrated that the presence of low-levels of triptolide greatly increased the cisplatin-induced apoptosis in A549 and HTB182 lung tumor cells. The results of our cell proliferation studies revealed that the presence of low-levels of triptolide only had a limited effect on cell proliferation of A549 and HBT 182 lung tumor cells. Therefore, it is unlikely that this increase in cisplatin-induced apoptosis is caused by a global transcription inhibition effect of triptolide as demonstrated from other studies [14]. The involving mechanism that causes this increased apoptosis of the lung cancer cells is unknown. Since triptolide is known to bind specifically to the XPB component of TFIIH basal transcription factor and TFIIH is required for both the transcription and NER processes, it is possible that inhibiting the NER process plays an important role in the mechanism through which the presence of low-level triptolide increases cisplatin-induced apoptosis in these lung tumor cells. The results of our reef coral red protein expression studies also provide very strong evidence to support this possibility: the presence of low-level triptolide only inhibited expression of reef coral red protein from cisplatin-damaged but not undamaged pDsRed2-C1 plasmid in A549 lung tumor cells. However, we cannot rule out a synergistic effect of both triptolide and cisplatin in this increased apoptosis. Further studies need to determine if both mechanisms contribute to the increased apoptosis or if only one of these mechanisms plays a major role in this increased apoptosis.

The results of our protein phosphorylation studies revealed that the presence of low-levels of triptolide caused a decrease in cisplatin-induced CHK1 phosphorylation at Ser^317/345^. Given the important role of CHK1 in both S- and G2-phase cell cycle arrests and in cell survival [19–22], it is likely that preventing DNA damage-caused CHK1 activation contributes to the mechanism through which triptolide increases cisplatin-induced apoptosis of these lung cancer cells. In addition, the results of our protein phosphorylation studies also revealed that the presence of triptolide caused a greater increase in cisplatin-induced ATM phosphorylation at Ser^1981^ in HTB182 lung tumor cells. The ATM phosphorylation at Ser^1981^ is caused by DNA double-stranded breaks (DSBs) [23] and ATM phosphorylation at Ser^1981^ causes activation of the ATM signaling pathway [18, 23]. Activation of the ATM pathway results in induced expression of important apoptotic proteins, such as PUMA [24, 25]. Therefore, it is possible that the presence of triptolide may also increase the cisplatin-induced apoptosis of A549 and HTB182 lung tumor cells by inhibiting the NER activity, which increases DSBs for cisplatin-treated cancer cells and causes activation of the ATM pathway.

Recent studies have demonstrated the anticancer activities of triptolide [11–13]. However, most of its anticancer activities were observed at high concentrations likely through inhibiting global transcription [11, 12, 14]. High-levels of triptolide are known to cause severely adverse effects, which reduces its application in cancer treatment. Our studies focused on testing the possibility of using low-levels of triptolide to selectively inhibit the NER activity, and therefore, to potentiate cancer cells to cisplatin-induced apoptosis while avoiding the adverse effects caused by high-levels of triptolide. The results of our studies demonstrated that the presence of low-levels of triptolide had little effect on cell proliferation or global transcription inhibition but had great effect in increasing cisplatin-induced apoptosis in both A549 and HTB182 lung tumor cells. In addition, the results of our reef coral red protein expression studies further revealed that the presence of low-levels of triptolide had little effect on expression of either reef coral red or GFP proteins from undamaged plasmids but had great effect on inhibiting expression of reef coral red protein from cisplatin-damaged pDsRed2-C1 plasmid in the A549 cells. These results suggest that the presence of low-levels of triptolide only inhibits the NER but not the transcription process. The mechanism through which the presence of low-levels of triptolide only selectively inhibits the NER but not the transcription process is unknown. One possibility is that two distinguished TFIIH sub-populations may exist and each has a unique role rather than playing dual roles in the NER and transcription processes. It is likely that these TFIIH may possess very different sensitivities to triptolide: the TFIIH involved in the NER process has a very high sensitivity to triptolide and is inhibited by low-levels of triptolide whereas the TFIIH involved in the transcription process has a much lower sensitivity to triptolide and is more resistant to triptolide treatment. In fact, some published works have already suggested this possibility: the TFIIH involved in the NER or transcription processes carries different protein components [26, 27]. It is possible that the triptolide binding site for the TFIIH involved in the NER process is more accessible for triptolide than the TFIIH involved in the transcription process, which makes the NER process more sensitive to the triptolide treatment than the transcription process. Alternatively, it is also possible that triptolide has other protein targets in addition to TFIIH. Since the effect of triptolide can be observed at relatively low concentration, it is suggesting higher affinity interaction between triptolide and its targets in the cell. Similar to the identification of Heat Shock Protein 90 (Hsp90) as the actual target of Geldanamycin, a potent anticancer agent that is currently under clinical development [28], it is possible to use reverse pharmacology to identify those possible additional target(s) of this promising anticancer agent by using triptolide or its analog. The further identification of the triptolide targeting protein(s) will provide potential novel targets for the development of more potent and specific anticancer drugs since triptolide has serious side effects at higher dose.

The results of our studies clearly demonstrated that the presence of low-levels of triptolide potentiated the A549 and HTB182 lung tumor cells to cisplatin-induced apoptosis. Given the wide application of cisplatin and other platinum-based drugs in cancer treatment and the great challenge of cancer cell resistance to platinum-based chemotherapy, our studies suggest a novel approach to overcome this cancer cell resistance and to improve the efficacy of platinum-based cancer treatment. Therefore, knowledge obtained from our studies will have important clinical relevance in cancer treatment, especially in platinum-based cancer treatment. However, further studies are needed to determine the clinical relevance of our studies before applying this knowledge clinically to cancer treatment.

Several studies have been published which demonstrate the presence of triptolide increased cisplatin-induced apoptosis in several cancer types, including bladder, gastric, liver, ovarian, and pancreatic cancer [29–34]. The involving mechanisms, however, have not been determined although some data has suggested the involvement of p53 [34] and NF-κB pathways [31] in the process. The results of our studies suggest that inhibiting the NER activity plays an important role for low-levels triptolide in increasing cisplatin-induced apoptosis of lung cancer cells. Therefore, our studies provide an important mechanism regarding the presence of low-level triptolide in increasing cisplatin-induced cancer cells apoptosis. This knowledge would have important implications on cancer treatment, especially in platinum-based cancer treatment.

Our studies reveal that the presence of low-levels triptolide can selectively inhibit NER activity without affecting transcription. These results not only suggest triptolide as a NER-specific inhibitor to enhance platinum-based cancer treatment, but also provide a very useful tool to study the mechanisms of DNA repair and transcription, especially the role of TFIIH in DNA repair and transcription. Therefore, the knowledge obtained from our studies not only has its clinical relevance in cancer treatment but also has its scientific importance in basic research, such as transcription and DNA repair.

## Materials and methods

### Cell lines, plasmids, and primers

The A549, HTB182, CRL5810, and CRL5922 lung tumor cells were purchased from the American Type Culture Collection (ATCC). The A549 lung tumor cells were derived from a lung carcinoma patient. The HTB182 lung tumor cells were derived from a lung squamous cell carcinoma patient. The CRL5810 lung tumor cells were derived from a stage-2 lung adenocarcinoma patient. The CRL5922 lung tumor cells were derived from a stage 1 lung adenocarcinoma patient. All the lung tumor cells were maintained in RPMI1640 medium supplemented with 10 % fetal bovine serum (FBS) at 37 °C with 5 % CO_2_.

The pDsRed2-C1 plasmid was purchased from Clonetech Laboratories, Inc. (Mountain View, CA). The pmaxGFP plasmid was purchased from Lonza Inc. (Anaheim, CA). The pDsRed2-C1 plasmid carried a CMV promoter-driving reef coral-red protein gene and the pmaxGFP plasmid carried a CMV promoter-driving green fluorescence protein (GFP) gene. Both pDsRed2-C1 and pmaxGFP plasmids were amplified in *E. coli* DH10B strain and purified using a QIAGEN Plasmid MaxiPrep Kit.

The real time PCR primers used in this study were listed in Table 1 and were synthesized by the Midland Certified Reagent Company (Midland, TX).

**Table 1:** Primers used in the real time PCR studies

1. Bcl_-XL_ primers:	
*Bcl*_*-XL*_ *forward primer:*	5’-GGTGAGTCGGATCGCAGCTTG-3’
*Bcl*_*-XL*_ *reverse primer:*	5’-CTCTCGGCTGCTGCATTGTTC-3’
2. Brca1 primers:	
*Brca1 forward primer:*	5’-CCAGCCTTCTAACAGCTACC-3’
*Brca1 reverse primer:*	5’-CTGGTAGAACTATCTGCAGAC-3’
3. Dnmt1 primers:	
*Dnmt1 forward primer:*	5’-GAGCAAGTCCGATGGAGAGGC-3’
*Dnmt1 reverse primer:*	5’-GATGGTGGTTTGCCTGGTGC-3’
4. Dnmt3a Primers:	
*Dnmt3a forward primer:*	5’-GATGAGCGCACAAGAGAGCG-3’
*Dnmt3a reverse primer:*	5’-CGTCGTACTGGTACGCACACTC-3’
5. Dnmt3b Primers:	
*Dnmt3b forward primer:*	5’-GGTGCGTCGTGCAGGCAGTAG-3’
*Dnmt3b reverse primer:*	5’-CTCGGCTCTGATCTTCATCC-3’
6. Puma Primers:	
*PUMA forward primer:*	5’-CTCGCTCTCGCTGGCGGAGCAG-3’
*PUMA reverse primer:*	5’-CGCTGCTGCTCTTGTCTC-3’
7. Xpg Primers:	
*Xpg forward primers*:	5’-GGAAGCTGCTGGAGTGCTCCG-3’
*Xpg reverse primers:*	5’-TGAGTTCCCATGGCGATCCCG-3’
8. β-Actin Primers:	
*β-Actin forward primer:*	5’-GTACGTTGCTATCCAGGCTGTG-3’
*β-Actin reverse primer:*	5’-CATGAGGTAGTCAGTCAGGTC-3’

### Chemicals

Both triptolide (from *Tripterygium wilfordii* with a purity ≥98 %) and cisplatin were purchased from Sigma Aldrich Inc. (St. Louis, MO). The triptolide was prepared as a 1 mg/ml stock in chloroform and stored at −80 °C. Cisplatin was prepared freshly in DMSO as a 50 mM solution and used immediately for the study. Because chloroform has limited solubility in water, the triptolide treatment was done by carefully adding the triptolide stock solution to the surface of cell culture medium and slowly shacking the dish until the solution completely dissolved into the medium.

### Cell proliferation assay

Both A549 and HTB182 lung tumor cells were seeded onto 100 mm dishes at the same cell number and incubated at 37 °C overnight to about 20 % confluence. The cell number was counted from one dish for both the A549 and HTB182 lung tumor cells before the triptolide treatment. Some of the dishes were then treated with triptolide (5 or 10 ng/ml) by carefully adding the triptolide stock solution to the surface of medium and slowly shacking the dishes until the solution completely dissolved into the medium. The cell number was counted for both the untreated and triptolide-treated cells from one dish for both the A549 and HTB182 cells at 24, 48, and 72 h after the triptolide treatment. The cell growth curve was generated for both the untreated and triptolide-treated A549 and HTB182 cells.

### Reverse transcription-based RNA quantification (real time PCR) assay

Total RNA was isolated from both untreated and triptolide-treated A549 (10 ng/ml triptolide) and HTB182 (5 ng/ml triptolide) cells 20 h after the treatment. A reverse transcription-based RNA quantitation (real time PCR) assay was performed to determine the mRNA levels of target genes using both the High Capacity cDNA Reverse Transcription kit (Applied Biosystems) and the Sybr Green-based Power PCR system (Applied Biosystems) with a StepOne Plus Real time PCR system (ABI). The level of β-actin mRNA was determined for each RNA sample and used as an internal control for RNA quantification. The mRNA levels of desired target genes in each RNA sample were calculated as fold changes in comparison to the same gene in the untreated A549 lung cancer cells.

### Caspase-3 activation assay

Cells were seeded onto 100 mm culture dishes and incubated at 37 °C overnight to approximately 30 % confluence. The cells were treated with triptolide and/or cisplatin at indicated concentrations by adding the reagents directly to the culture medium and incubated at 37 °C incubator for 26 h (CRL5810 and CRL5922) or 36 h (A549 and HTB182). The cells were collected and re-suspended in 300 μl insect cell lysis buffer (BD Pharmagen). The cells were lysed by using sonication for 2 s and then centrifuged at 4 °C for 10 min. The supernatants were collected and the caspase-3 activity was measured from each lysate using a protocol described in our previous study [6, 35].

### DNA repair-mediated reef coral-red protein expression assay

The pDsRed2-C1 plasmid DNA was incubated with 10 μM cisplatin in 10 mM Tris (pH8.5) at 37 °C for 6 h to generate cisplatin DNA damage into the plasmid DNA and then precipitated by 70 % ethanol to remove free cisplatin. The cisplatin-damaged plasmid DNA was then dissolved into 10 mM Tris (pH8.5) and used for the reef coral-red protein expression study. For transfection, the A549 cells were harvested and re-suspended in Opti-MEM medium at a density of 4×10^6^ cells/ml. The A549 cells (3×10^6^ cells) were incubated with both pDsRed2-C1 (4 μg of either undamaged or cisplatin-damaged plasmid DNA) and pmaxGFP (2 μg) plasmid DNA at room temperature for 5 min and then transfected by electroporation in 0.4 cm cuvettes with a setting of 250v/25 μF. The cells were incubated at room temperature for 30 min and then seeded onto two 100 mm cell culture dishes. One dish was treated with triptolide (10 ng/ml) whereas the other dish remained untreated. The cells were cultured at 37 °C incubation for 24 h and expressions of both reef coral-red and GFP proteins were visualized by fluorescence microscope using the excitation/emission light wavelengths of 563 nm/582 nm and 475 nm/505 nm for reef coral-red and GFP proteins respectively. The black-white images were also documented for the same view for visualization of the cells.

### Western blotting assay

Cell lystates (20 μg total protein) prepared from both untreated and treated A549 and HTB182 lung tumor cells were analyzed by western blotting to determine the levels of CHK1 phosphorylation at Ser^317/345^ and ATM phosphorylation at Ser^1981^ using antibodies that recognized the CHK1 phosphorylation at Ser^317^ and Ser^345^ and ATM phosphorylation at Ser^1981^ (Cell Signaling). The protein level of tubulin was also determined for the cell lysates as a protein loading control in the same membrane.

### Statistical analysis

All data was expressed as the Mean ± standard deviation (S.D.). Statistical analysis was done using a GraphPad Prism software (La Jolla, CA). Statistically significant differences were determined using a student *t*-test with 95 % confidence interval (CI). The data was obtained from at least three independent experiments.
